# 

**Published:** 1913-02-15

**Authors:** 


The twentieth annual meeting of the Hostel of St. Luke will be held in the Church House, Dean's Yard, Westminster, on Monday, February 17, 1913, at four o'clock. The chair will be taken by the Rt. Rev. the Lord Bishop of Gloucester, V.P., who will be supported by the Rt. Rev. Bishop E. N. Powell, D.D., Sir Alfred Cripps, K.C.V.O., K.C., Miss C. H. Mayers, Arthur H. Tritton, Esq. (chairman and hon. treasurer), and other members of the Council and of the Executive and Medical Committees.

